# The Combined Effects of *Varroa destructor* Parasitism and Exposure to Neonicotinoids Affects Honey Bee (*Apis mellifera* L.) Memory and Gene Expression

**DOI:** 10.3390/biology9090237

**Published:** 2020-08-20

**Authors:** Nuria Morfin, Paul H. Goodwin, Ernesto Guzman-Novoa

**Affiliations:** School of Environmental Sciences, University of Guelph, 50 Stone Road East, Guelph, ON N1G 2W1, Canada; pgoodwin@uoguelph.ca (P.H.G.); eguzman@uoguelph.ca (E.G.-N.)

**Keywords:** honey bees, learning, memory retention, neonicotinoids, clothianidin, *V. destructor*

## Abstract

Honey bees (*Apis mellifera* L.) are exposed biotic and abiotic stressors but little is known about their combined effect and impact on neural processes such as learning and memory, which could affect behaviours that are important for individual and colony survival. This study measured memory with the proboscis extension response (PER) assay as well as the expression of neural genes in bees chronically exposed to three different sublethal doses of the insecticide clothianidin and/or the parasitic mite *Varroa destructor*. The proportion of bees that positively responded to PER at 24 and 48 h post-training (hpt) was significantly reduced when exposed to clothianidin. *V. destructor* parasitism reduced the proportion of bees that responded to PER at 48 hpt. Combined effects between the lowest clothianidin dose and *V. destructor* for the proportion of bees that responded to PER were found at 24 hpt. Clothianidin, *V. destructor* and their combination differentially affected the expression of the neural-related genes, *AmNrx-1* (*neurexin*), *AmNlg-1* (*neuroligin*), and *AmAChE-2* (*acetylcholinesterase*). Different doses of clothianidin down-regulated or up-regulated the genes, whereas *V. destructor* tended to have a down-regulatory effect. It appears that clothianidin and *V. destructor* affected neural processes in honey bees through different mechanisms.

## 1. Introduction

Honey bees (*Apis mellifera* L.) have a complex social organization in which worker bees perform a number of behaviors that are essential for colony survival [1]. To perform behaviors, bees rely on neural processes that allow them to react to environmental stimuli after perceiving and processing them through the central nervous system [2]. Neural processes are indispensable for behaviors that require associative learning, such as foraging behavior, in which bees remember the location of a food source and its distance to the hive using landmark maps and odor cues. This information is communicated to nestmates through the waggle dance [3,4]. Thus, associative learning is an essential neural process in honey bees.

Associative learning in honey bees can be affected by a number of factors, including neurotoxins [5]. Neurotoxins interact with neural receptors, ion channels and signaling pathways, which in turn may result in behavioral impairment [6]. Neonicotinoids, the most widely used insecticides worldwide, are neurotoxins [7,8], and clothianidin is one of the most commonly used neonicotinoids in field crops, particularly in corn and canola [9]. Thus, non-target insects, such as honey bees, could be exposed to repeated sublethal doses of neonicotinoids like clothianidin by foraging in plants with contaminated nectar and pollen [5,10]. The neurotoxic effect of neonicotinoids is related to their affinity for acetylcholine receptors (AChR) acting as agonists on nicotinic acethylcholine receptors (nAChRs), inducing a continuous opening of ion channels in the neurons, which leads to an excitatory state in the insect’s nervous system [11,12]. Neonicotinoids have been reported to negatively affect bee learning and memory [13].

Associative learning in honey bees can also be affected by parasites [5]. One of the major parasites of honey bees is ectoparasitic mite, *Varroa destructor. V. destructor* parasitism has been associated with overwinter colony mortality [14,15]. At the individual level, *V. destructor* reduces longevity, causes weight loss, impairs homing ability and immunosuppresses honey bees [16,17,18,19,20]. Additionally, *V. destructor* acts as a vector for viruses that adversely affect the health of honey bees, mainly the deformed wing virus (DWV) [21,22]. Moreover, *V. destructor* has been reported to affect non-associative learning [23], and DWV impairs associative learning [24].

One of the most commonly used assays to measure associative learning and memory retention in bees is the proboscis extension response (PER) assay [25]. The PER assay consists of presenting an odor (conditioned stimulus, CS) along with a sugar reward (unconditioned stimulus, US) to stimulate the extension of the proboscis of a bee. Memory retention is tested by presenting the CS to the bee and record if she is able to extend her proboscis once the bee learns to associate the CS with the US [26]. Using the PER assay, it has been reported that neonicotinoids may affect memory and learning [27,28,29,30,31]. However, most of those studies have focused on single acute neonicotinoid exposure or treated bees *ad libitum* with sublethal doses of clothianidin for only 24 h [32,33,34], rather than multiple exposures to sublethal doses of neonicotinoids or the interaction with other stressors on the bees known to affect memory and learning, like parasites.

Among the factors that affect associative learning and memory in honey bees are neurotransmitters and synaptic proteins [35]. Acetylcholine (ACh) is one of the main neurotransmitters of the central nervous system and has been shown to be involved in learning processes in honey bees [36,37]. Thus, the expression of the gene for acetylcholinesterase (*AChE*), which encodes the enzyme for the catalytic breakdown of ACh, could have an important role in learning and memory in honey bees [38]. Additionally, pre and post-synaptic proteins, such as neurexins and neuroligins [39], have been recognized to be important in associative learning honey bees. The expression of the genes for neurexin (*AmNrx-1*) and neuroligin (*AmNlg-1*) were up-regulated in bees following PER training compared to non-trained control, indicating that they are affected by changes associated with learning and memory retention [40]. As expression of *AmNrx-1, AmNlg-1* and *AmAChE-2* has been previously used as molecular markers to assess the effect of stressors on neural gene expression [41], they could also be informative markers for studying the effects of stressors and their combined effects on honey bee memory retention.

Because neural processes regulate and influence honey bee behaviors affected by memory that are essential for colony survival, the aim of this study was to evaluate the impact of field-realistic repeated exposure to sublethal doses of clothianidin, *V. destructor* parasitism and the combined effect of clothianidin and *V. destructor* parasitism on memory retention and expression of *AmNrx-1, AmNlg-1* and *AmAChE-2* in honey bees. DWV levels were also determined.

## 2. Materials and Methods

### 2.1. Source of Honey Bees and V. destructor Mites

Bees were obtained from colonies of the Buckfast strain bred at the Honey Bee Research Centre, University of Guelph, Ontario, Canada. The queens of colonies that produced the workers used in this study were mated under controlled conditions in isolation at Thorah Island, Simcoe, Ontario. The colonies were not subjected to any treatment or exposed to pesticides at any time during the study. Female *V. destructor* mites were collected from infested colonies as per Arechavaleta-Velasco and Guzman-Novoa [42], and placed in Petri dishes for their immediate use in the experiments.

### 2.2. Sublethal Doses of Clothianidin

The sublethal field realistic doses of clothianidin used in the study were calculated based on estimates of the amount of nectar a bee consumes in a day (25.5–39 mg) [1], as well as on the concentration of clothianidin found in nectar of canola grown from seeds treated with the insecticide (0.0012–0.0086 ng/mg [43,44]. Based on these calculations an adult bee could consume between 0.03 and 0.34 ng of clothianidin per day ($\bar{x}$ = 0.15 ± 0.06 ng). The concentration of clothianidin (ng/µL) was calculated considering a daily consumption of 30–33 µL of 50% sucrose syrup per bee per day [45,46]. To prepare the doses of the insecticide, 10 mg clothianidin (Sigma Aldrich, Oakville, ON, Canada) was dissolved in 100 mL double distilled (ds) H_2_O, and then serial dilutions were made in 50% sucrose syrup to obtain 9 × 10^−4^ ng clothianidin/µL, 4.2 × 10^−3^ ng clothianidin/µL, and 1 × 10^−2^ ng clothianidin/µL. A control was 0 ng clothianidin/µL.

### 2.3. Exposure to Clothianidin and/or V. destructor

Newly emerged bees were obtained from the source colonies and managed as described by [41,47]. There were seven treatments of clothianidin with or without *V. destructor* along with a non-treated control listed in Table 1. For each repetition, 20 to 40 bees were randomly assigned to each treatment and placed in hoarding cages (12.7 × 8.5 × 14.5 cm) that were kept in an incubator (32 ± 2 ˚C, 50 ± 10% RH) for 14 days. Each cage was provided with one 20 mL gravity feeder containing 50% sucrose syrup treated or not with different concentrations of clothianidin. To determine the amount of syrup consumed by the bees (and thus the amount of clothianidin), the feeders with the syrup were weighed before providing them to the cages and on days 3, 7 and 14, using a balance (Denver Instruments Model S-403, Bohemia, NY, USA). For the four treatments that had bees parasitized with *V. destructor*, a female mite was placed on the body of each bee using a fine paintbrush, and the attachment of the mite to the bee’s body was visually confirmed. The continued attachment of *V. destructor* over the 14 day period before the PER assays was not monitored, as *V. destructor* mites move, re-infest other bees or die. Bee mortality was recorded daily during the 14 days that the treatment lasted. The bees were treated during 14 days prior to the PER assays to ensure a chronic exposure to the stressors, and also because olfactory memory is age dependent, with bees between 13 and 16 days of age showing good memory retention in PER assays [48]. Seven repetitions of this experiment were conducted and the treatments that belonged to the same biological repetition were performed simultaneously.

### 2.4. Effect of Sublethal Doses of Clothianidin and/or V. destructor on Memory Retention

Memory retention was evaluated using the PER assay [49], which consists in training the bees to associate an odor as CS with a sugar reward as US. Briefly, each bee was immobilized by carefully introducing her into a modified 1.5 mL microcentrifuge tube (to which a 4 mm cut was made at the bottom) in an upright position so that the head was exposed and the bee could freely move her head and proboscis, but not the rest of the body. A piece 1 × 2 cm piece of lab wipe (Kimwipes, Fisher Scientific, Mississauga, ON, Canada) was placed at the bottom of the tube to help the bee maintain its position. Each bee was assigned an identification number before the PER assay. Groups of 15 to 20 bees per treatment were kept in their respective tubes placed upright in a plastic microcentrifuge tube rack (Fisher Scientific, Mississauga, ON, Canada). After placing the bees in the rack, they were not disturbed for 30 min to allow them to acclimatize before being fed with 5 µL of 50% sucrose syrup using a micropipette (Fisherbrand, Mississauga, ON, Canada). The bees were then placed in a dark room at room temperature (20 to 22 °C) for 24 h before initiating the PER assay. The bees were individually fed 33 µL of 50% sucrose syrup (without clothianidin), after the training or memory tests, once a day at 16:00 h EST over the three days that the PER assay lasted.

First, each bee was trained to associate the CS with the US as per Felsenberg et al. [49]. A restrained bee was placed 3 cm from the tip of a syringe, which was used to deliver the scented air (CS). To produce scented air, the syringe barrel contained a 19.5 mm diameter piece of Whatman™ filter paper (Fisher Scientific) impregnated with 5 µL of clove oil (*Eugenia* spp.; Sigma-Aldrich). An aluminum airduct (100 mm diameter, 1000 mm long) was placed at the opposite side of the bee with a fan at the end of the airduct to suck the air through the airduct away from the syringe tip, thus preventing the clove oil scent from remaining at the station after exposure to the bee.

Each bee was exposed to clove oil scent during 10 s continuously by gently pushing the syringe plunger to deliver the scented air through the syringe tip. After the initial 3 s of exposure, the bee’s antennae were touched with a toothpick impregnated with sucrose syrup for 3 s to stimulate the proboscis extension response. Only if the bee extended the proboscis was she allowed to taste the syrup by her proboscis for the last 4 s of odor exposure. The toothpick was withdrawn for 4 s, and then the bee was again exposed to the toothpick to taste the sugar syrup for 3 additional s to reinforce learning the US [50]. Three training trails were performed per bee with intervals of 10 min between trails.

Each bee was subjected to three memory retention tests at 2, 24 and 48 h post-training (hpt). To test memory retention, each bee was exposed to clove oil scent for 5 s as described before, and the extension of the proboscis was recorded (positive or negative event [51]). After each PER assay was concluded, a stimulus response (SR) test was performed by presenting a toothpick with sucrose syrup to the bees’ antennae to verify that the bee was able to extend the proboscis indicating that no damage was suffered during the assay [52]. The bees that could not extend their proboscis during the SR test were not included in the results, and only the bees that survived until the last day of the PER assay were included in the statistical analyses. Seven repetitions were conducted for the PER assay, and treatments that belonged to the same biological repetition were performed simultaneously. From a total of 1463 bees that initiated the PER assay, only 864 were included in the statistical analyses for learning and memory. After the completion of the last PER assay, the bees were frozen at −70 °C for further analyses.

### 2.5. RNA Extraction and cDNA Synthesis

Total RNA was extracted from six to eight bees (full bodies) from each of three biological repetitions using TRIzol^®^ Reagent (Fisher Scientific) following the manufacturer’s instructions. The quality and concentration of the RNA were measured by determining the OD 260/280 nm ratio using a spectrophotometer (NanodropLite, Thermo Scientific, Mississauga, ON, Canada). Values between 1.8 and 2.0 were considered acceptable for purified RNA. cDNA was prepared using a RevertAid H Minus First Strand cDNA Synthesis Kit (Fermentas, Burlington, ON, Canada) following the manufacturer’s instructions and using 2000 ng of RNA for each sample. The cDNA was stored at −20 °C.

### 2.6. DWV Quantification

To calculate the number of DWV genome copies (gc) per sample, primers specific for the DWV helicase and the PCR conditions of Di Prisco et al. [53] were used. Absolute quantification was performed using a BioRad CFX96™ thermocycler (Bio-Rad Laboratories, Mississauga, ON, Canada) and PowerUp Sybrgreen (2×) (Thermo Scientific). Each reaction consisted of 2 µL template, 0.4 µL forward and reverse primers (200 nM final concentration), 10 µL PowerUp Sybrgreen (2×) and 7.2 µL nuclease-free H_2_O. As a negative control, nuclease-free H_2_O was included instead of cDNA, and a positive control from previously identified DWV positive samples by qRT-PCR were included in each qRT-PCR run. Calibration curves to convert Ct values to DWV genome copies were performed using 300 bp gBlocks (Integrated DNA Technologies, Coralville, IA, USA) that included the sequence of the forward primer, amplicon and reverse primer. The lyophilized gBlock was diluted with 20 µL of ds H_2_O to obtain an initial concentration of 10 ng/µL that was used to make serial dilutions from 109 to 101 copies. A linear equation was used to calculate the DWV genome copy numbers for each of the samples using the Ct values for each sample and known DWV copy number used to produce the standard curves. Three technical repetitions were performed for each of the three biological repetitions for DWV quantification.

### 2.7. Quantitative Real Time (qRT-PCR) and Gene Expression Analysis

The qRT-PCR was performed with a BioRad CFX96 thermocycler (Bio-Rad Laboratories) with PowerUp™ Sybrgeen (2×) (Thermo Scientific). Reactions were performed in 20 µL: 2 µL template, 0.6–1.4 µL primers (primer concentration varied between 400 and 700 nM, depending on the target optimization protocol), 10 µL PowerUp Sybrgreen (2×), and 5.2–6.8 µL nuclease-free H_2_O. The PCR conditions consisted of one cycle at 50 °C for 2 min, one cycle at 95° C for 10 min and 40 cycles at 95 °C for 15 s, followed by 60 s at 60 °C. To confirm the specificity of the target gene, a melt curve analysis was included after each qRT-PCR run. Among candidate constitutive genes (*β-actin*, *AmRPS5* and *AmGAPD2* [53,54,55]), *AmRPS5* was selected as the reference gene as it had the lowest stability value at 0.13 compared to the stability value of *β-actin* (0.26) and *AmGAPD2* (0.27), as determined by NormFinder [56]. Primers for *AmRPS5* were those used by Evans [54], *AmNrx-1* by Morfin et al. [57], *AmNlg-1* by Biswas et al. [40], and *AmAChE-2* by Morfin et al. [41].

The expression level of the target gene was normalized to the expression level of the reference gene using the 2^−ΔΔCt^ (Livak) method [58] with the non-treated control group as calibrator. The Bio-Rad CFX Manager^®^ software (Bio-Rad Laboratories) was used to calculate the expression ratio. Three technical repetitions were performed for each of the three biological repetitions for the analyses of gene expression.

### 2.8. Statistical Analyses

To analyse the probability of survival, the data of surviving bees were subjected to the Kaplan–Meier log rank method and the curves were compared using pairwise comparisons with an adjusted *p*-value and log rank (Mantel–Cox) tests. PER data from the training trials and memory retention tests were analysed with contingency tables using Chi2 tests of independence and adjusted residuals were calculated for post-hoc analysis to determine whether the proportion of bees positive to PER were different between treatments. The data for relative gene expression were tested with a Shapiro–Wilk test and were log_2_ transformed due to lack of normality. The transformed data were again subjected to Shapiro–Wilk and Levene tests to confirm normality and equality of variances before being subjected to a two-way ANOVA and Tukey HSD tests. The data on sugar consumption and DWV quantification were subjected to Kruskal–Wallis tests and Conover–Iman procedure, as they did not comply with normality. The above statistical analyses were performed using R studio version 3.4.3 [59] and IBM SPSS Statistic 25 [60] with the significance level set at *p* < 0.05 (α of 0.05).

## 3. Results

### 3.1. Survival and Sugar Consumption

**Figure 1:**
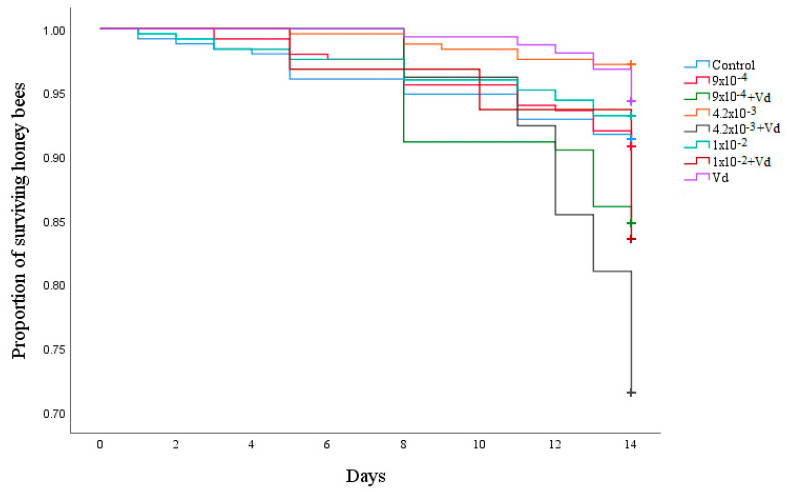
Kaplan–Meier survival curves of adult bees exposed to field realistic doses of clothianidin (0 ng clothianidin/µL, 9 × 10^−4^ ng clothianidin/µL, 4.2 × 10^−3^ ng clothianidin/µL, and 1 × 10^−2^ ng clothianidin/µL) and/or *V. destructor* (Vd) for 14 consecutive days.

No significant differences in the survival curves of the different treatments were found (χ^2^_(1)_ = 0.34, *p* = 0.55; Figure 1).

There was no significant effect of the treatments on sucrose syrup consumption (H_(7)_ = 9.75, *p* = 0.20; Table 1).

### 3.2. Memory Retention

**Figure 2:**
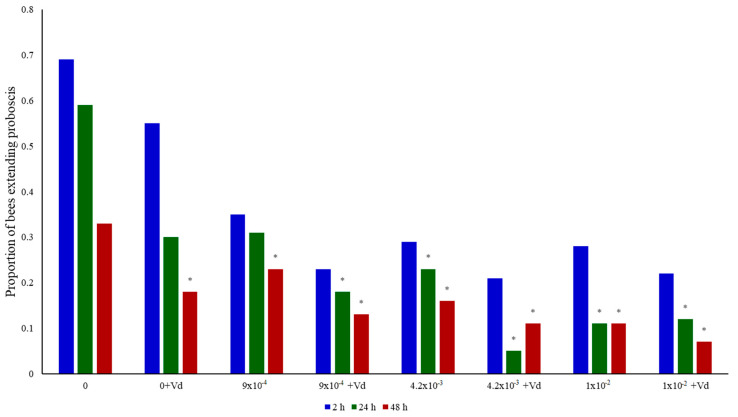
Proportion of bees positive for memory retention at 2, 24 and 48 hpt. The bees were treated with clothianidin (0 ng clothianidin/µL, 9 × 10^−4^ ng clothianidin/µL, 4.2 × 10^−3^ ng clothianidin/µL, and 1 × 10^−2^ ng clothianidin/µL) and/or *V. destructor* (Vd) before the first training trial. The asterisks indicate a significant reduction in the proportion of bees positive to memory retention compared to the controls based on Chi^2^ analyses and adjusted residuals (α of 0.05).

Significant treatment effects were found for the proportion of bees positive for memory retention in the PER assay (Chi^2^_(23)_ = 382.96, *p* < 0.0001; Figure 2 and Supplementary Table S1). The post-hoc analysis showed no effects on the proportion of bees positive for memory retention at 2 hpt (*p* > 0.05). However, the medium and highest doses of clothianidin significantly reduced the proportion of bees positive for memory retention at 24 hpt (*p* < 0.05). *V. destructor* alone did not have an effect on the proportion of bees positive memory retention at 24 hpt (*p* > 0.05), but it did significantly decline in parasitized bees treated with clothianidin, regardless of the dose (*p* < 0.05). Thus, it seems that clothianidin combined with *V. destructor* have a negative effect on memory retention at 24 hpt. A significant reduction in the proportion of bees positive to memory retention was noted in bees exposed to the three doses of clothianidin, *V. destructor* or the combined stressors at 48 hpt (*p* < 0.05), indicating that both stressors affected long-term memory.

### 3.3. DWV Levels

**Figure 3:**
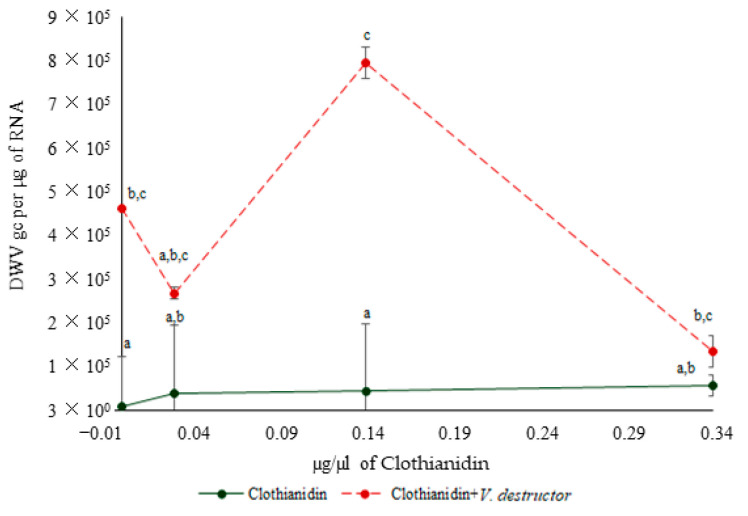
Mean DWV genome copies (GCs) per µg of RNA (± S.E.) of bees that were exposed to clothianidin and/or *V. destructor*. Different letters (a, b and c) above the bars indicate significant differences based on a Kruskal–Wallis test and Conover Iman procedure. Non-transformed data are presented.

There was a significant effect of the treatments on DWV levels (H_(7)_ = 29.99, *p* < 0.0001; Figure 3). A significant increase in DWV gc was observed in bees treated with 4.2 × 10^−3^ ng/µL of clothianidin plus *V. destructor* compared to the control (*p* < 0.05). Bees parasitized by *V. destructor* showed DWV gc that was 11-fold higher than that of non-parasitized bees. The bees exposed to 4.2 × 10^−3^ ng/µL of clothianidin plus *V. destructor* had 17-fold higher DWV gc than the bees exposed to the same dose of clothianidin alone. However, there were no significant differences on DWV levels between the bees exposed to clothianidin alone and the control (*p* > 0.05). Thus, *V. destructor* was the main factor associated with an increase in DWV levels.

### 3.4. Gene Expression in Bees Assessed for Memory Retention

**Figure 4:**
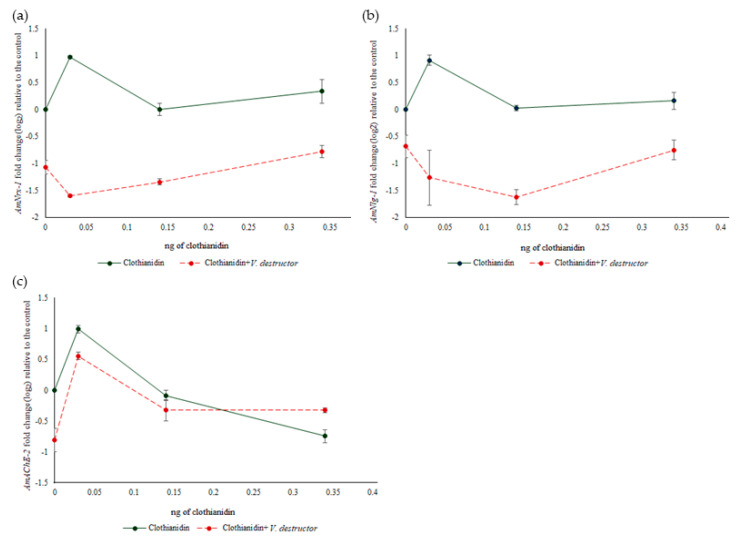
Mean (± SEM) relative expression versus ng of clothianidin of bees positive to PER of the genes: *AmNrx-1* (**a**); *AmNlg-1* (**b**); *AmAChE-2* (**c**). The relative gene expression was calculated using the Livak 2^−ΔΔCt^ method, with *AmRPS5* as reference gene and 0 ng as calibrator. Log_2_ transformed data are presented.

*AmNrx-1* expression was significantly affected by clothianidin treatments (F_(3,16)_ = 7.190, *p* = 0.003) and *V. destructor* (F_(1,16)_ = 396.865, *p* < 0.0001), and clothianidin treatments interacted with *V. destructor* (F_(3,16)_ = 21.313, *p* < 0.0001; Figure 4a). Compared to 0 ng/µL of clothianidin, significant differences were a 1.0 log_2_-fold up-regulation with 9 × 10^−4^ ng/µL of clothianidin alone (*p* < 0.0001), as well as a 0.78 to 1.6 log_2_-fold down-regulations by *V. destructor* alone (*p* < 0.0001), 9 × 10^−4^ ng/µL of clothianidin plus *V. destructor* (*p* < 0.0001), 4.2 × 10^−3^ ng/µL of clothianidin plus *V. destructor* (*p* < 0.0001) and 1 × 10^−2^ ng/µL of clothianidin plus *V. destructor* (*p* = 0.002). *AmNrx-1* expression was always lower with *V. destructor* parasitism than without it although expression increased as higher doses of clothianidin were included with *V. destructor*. However, there was always a significant difference in the expression of this gene in bees treated with clothianidin plus *V. destructor* compared to the corresponding doses of clothianidin alone (*p* < 0.0001, *p* < 0.001 and *p* < 0.0001, respectively). Overall, the main effect on *AmNrx-1* expression was shown as an up-regulation by the lowest dose of clothianidin, and a down-regulation of the gene in bees parasitized with *V. destructor*.

*AmNlg-1* expression was not significantly affected by clothianidin treatments (F_(3,16)_ = 2.016, *p* = 0.15), but was significantly affected by *V. destructor* (F_(1,16)_ = 49.775, *p* < 0.0001), and an interaction between clothianidin treatments and *V. destructor* was observed (F_(3,16)_ = 8.038, *p* = 0.002; Figure 4b). The expression patterns of *AmNlg-1* was very similar to that of *AmNrx-1* with treatments, except that expression declined from 9 × 10^−4^ ng/µL clothianidin plus *V. destructor* to 4.2 × 10^−3^ ng/µL clothianidin plus *V. destructor* with *AmNlg-1*, whereas it increased with *AmNrx-1*. The only significant difference relative to 0 ng/µL clothianidin between *AmNlg-1* and *AmNrx-1* expression was that the down-regulation by *V. destructor* alone was significant for *AmNrx-1* expression (*p* < 0.0001) but not for *AmNlg-1* expression (*p* = 0.37).

*AmAChE-2* expression was significantly affected by clothianidin treatments (F_(3,16)_ = 40.402, *p* < 0.0001) and *V. destructor* (F_(1,16)_ = 8.119, *p* = 0.012), and an interaction between clothianidin treatments and *V. destructor* was observed (F_(3,16)_ = 7.616, *p* = 0.002; Figure 4c). The pattern of expression of this gene with clothianidin alone showed an up-regulation in bees treated with 9 × 10^−4^ ng of clothianidin followed by a decrease in the gene’s expression as the dose increased. The pattern of *AmAChE-2* expression in bees exposed to clothianidin plus *V. destructor* also showed a similar dose response, but with no changes with the medium and highest doses of clothianidin plus *V. destructor*. Compared to 0 ng/µL clothianidin, significant differences were observed for a 1.0 log_2_-fold up-regulation in bees treated with 9 × 10^−4^ ng of clothianidin alone (*p* < 0.0001), 0.80 log_2_-fold down-regulation in bees exposed to *V. destructor* alone (*p* = 0.003), and 0.55 log_2_-fold up-regulation in bees treated with 4.2 × 10^−3^ ng of clothianidin plus *V. destructor* (*p* = 0.04). Thus, the lowest dose of clothianidin had an up-regulatory effect on *AmAChE-2*, *V. destructor* alone had a down-regulatory effect, and the effect of the interaction between the two stressors was due to *V. destructor* reducing the down-regulatory effect of the highest dose of clothianidin.

## 4. Discussion

No effects of the treatments on survival were observed in this study. The results on mortality agree with those of Morfin et al. [47] who reported less than 2% mortality for bees that received the same treatments as in this study but over 7 days. Conversely, Morfin et al. [41] reported a decrease in the probability of survival in *V. destructor* parasitized bees, and in bees exposed to clothianidin (using the same doses as in this study), but for a longer period of exposure (21 days versus 14 days in this study and 7 days for Morfin et al. [47]), and concluded that the major stressor associated with decreased survival was *V. destructor*. Thus, it appears that longer periods of parasitism results in lower probability of survival with or without clothianidin. The results on the effect of clothianidin on survival also agree with those of previous studies that found no effect on survival with chronic sublethal exposure to neonicotinoid insecticides [29,61,62]. However, at a colony level, Straub et al. [63] found a decrease in overwinter colony survival with sublethal doses of thiamethoxam, clothianidin and *V. destructor*, whereas Siede et al. [64] reported no effects of clothianidin and *V. destructor* on colony mortality and overwinter success. The results could differ due to the many differences in methodologies as well as the concentrations of neonicotinoid used.

This study found no effects of the stressors on sugar consumption. Similarly, Tosi et al. [62] showed that sugar consumption was unaffected in bees exposed to sublethal doses of clothianidin. While food consumption was reduced in bees exposed to sublethal doses of imidacloprid [65], those doses were 10-fold higher than the oral LD_50_ (17.32 ng/bee) [66] and also much higher than used in this study.

Honey bees depend on neural processes, such as learning and memory, to perform behaviours that are necessary for their survival, like foraging, flying to congregation areas to mate or when performing hygienic tasks [67]. Thus, a reduction in a bees’ memory could have negative consequences for the colony. This scenario could occur when honey bees are exposed to stressors like neurotoxic insecticides and pathogens. The present study revealed a negative effect of the highest dose of clothianidin, with or without *V. destructor,* on memory retention. Further, the study showed that not only does sublethal exposure to clothianidin and *V. destructor* parasitism negatively affect memory retention in honey bees, but it also is the first to show an effect of the two stressors combined on memory retention. None of the stressors affected survivorship or memory retention in the short term (2 hpt), but two doses of clothianidin affected it in the midterm (24 hpt), and an effect between the lowest dose of clothianidin plus *V. destructor* was also observed as a decrease in the proportion of bees positive to memory retention at 24 hpt. The greatest impact was on long-term memory (48 hpt) where both stressors, alone or combined, significantly reduced memory retention. However, the effect of clothianidin on olfactory processing instead or in addition to memory retention could not be discarded, as an effect of neonicotinoids on olfactory response has been reported [34,68].

The effects of sublethal exposure to clothianidin on honey bee learning and memory found in this study partially agree with previous studies, which used a similar experimental protocol. For example, Alkassab and Kirchner [69] reported memory retention impairment in adult bees treated with clothianidin in sucrose syrup administered *ad libitum* for 12 consecutive days, exposing the bees to a lower dose (2.6 × 10^8^-fold lower than the LD_50_) than those used in this study. While memory retention tested by PER was not affected at 1 hpt or 24 hpt, the specificity of memory (i.e., which among two types of odor was detectable) was significantly reduced at 24 hpt. Thus, it appears that the dose and time after the last training trial impacted memory retention. However, Alkassab and Kirchner [69] reported no significant effects of sublethal chronic exposure to clothianidin on learning during six training trials, which could be related to their use of lower doses and older bees (overwintered bees versus newly emerged bees in this study) for the PER assays. Piiroinen and Goulson [70] also reported negative effects on olfactory learning and memory in bees fed *ad libitum* with sucrose syrup containing clothianidin (3 × 10^2^-fold lower than the LD_50_) for 12 consecutive days, which is similar to the doses and mode of exposure used in this study. Moreover, Piiroinen and Goulson [70] used the PER assay to test the interaction of clothianidin and *Nosema,* and found impairment in learning in honey bees infected with *Nosema* spores and in bees exposed to clothianidin alone, but no interaction was observed between clothianidin exposure and *Nosema* infection. While a combined effect of clothianidin and *V. destructor* was found in this study, it is difficult to compare the studies considering that *Nosema* is an intestinal parasite that damages the midgut epithelial cells [71] and *V. destructor* is an ectoparasite that feeds upon the fat body and hemolymph, and transmits viruses to honey bees [72,73,74]. It may be that different pathogens interact differently with clothianidin, impacting neural processes or not.

There are few reports on the effect of parasites on associative learning in bees. Contrary to this study, Kralj et al. [23] did not find differences for associative learning using the PER assay between parasitized and not parasitized bees with *V. destructor*. However, Kralj et al. [23] evaluated memory retention only at 1 and 12 min after training, whereas this study assessed memory retention at 2, 24 and 48 hpt and no effects were observed at 2 hpt. Therefore, effects at 1 and 12 min after training are unlikely. In addition, Kralj et al. [23] used adult bees parasitized for only one day compared to using adult bees parasitized for 14 days in this study. Based on these differences, it is not surprising that the results of the studies differ. However, Zanni et al. [75] reported negative effects on associative learning and memory in bees parasitized by *V. destructor* at the 5th instar larval stage, subjected to six training trials, and evaluated for memory retention 22 to 24 days after they were parasitized. Thus, the effect of *V. destructor* on associative learning and memory retention could be linked to the chronicity of the parasitism, like in this study. Allowing for a longer period, however, means that the effect could not only be due to *V. destructor* parasitism, but also to the effects of viruses that are vectored by *V. destructor* as they can infect the central nervous system of bees [21,76]. This study found that bees parasitized by *V. destructor* had higher levels of DWV than non-parasitized bees, as has previously been reported [77,78,79], and this could have affected memory retention. DWV can rapidly replicate, increasing from 10^1^ to 10^4^ gc per bee in seven days after exposure to *V. destructor* [80], and thus 14 days would have allowed time for considerable replication. Iqbal and Mueller [24] found that bees artificially inoculated with DWV three days before training showed impaired learning and memory as measured by the PER assay at 2 and 24 hpt, and three days should have been enough time for DWV to replicate in the brain tissue. Hence, there was sufficient time in this study for DWV to multiply in the experimental bees, causing detrimental effects on memory retention. In addition, *V. destructor* parasitism could impair memory by reducing the energy supplies of the bees (glycogen and triglycerides) after reduction in fat body or the loss of hemolymph by mite parasitism [16,74,81]. A reduction in energy supplies could also affect the activation of cellular immune responses, as the activation of defense mechanisms has an energetic cost for the hosts and could decrease the availability of energy to perform other functions or behaviors, such as learning [20,82]. Clearly, the effect of *V. destructor* parasitism and viral infection on memory retention could not be differentiated in this study. Further studies are warranted to study the separate effects of each of these stressors.

*AmNrx-1* and *AmNlg-1* expression was examined in this study as they appear to be markers of the effects of clothianidin exposure [41]. The expression of *AmNrx-1* and *AmNlg-1* showed a similar pattern in bees exposed to clothianidin alone with significant up-regulation of *AmNrx-1* with the lowest dose of clothianidin alone, possibly related to the neuro-stimulatory effect of neonicotinoids at low doses [83]. In contrast, *V. destructor* had a down-regulatory effect on their expression, and there was a significant effect when combining the two stressors. Since their up-regulation could be associated with PER response, the down-regulation of *AmNrx-1* and *AmNlg-1* by *V. destructor* alone or combined with clothianidin could reflect a negative impact of those treatments on memory retention, which is consistent with the decreased proportion of bees being positive for long-term memory at 48 h with those treatments. However, Morfin et al. [41] found that *AmNrx-1* and *AmNlg-1* expression was up-regulated by the medium and highest doses of clothianidin plus *V. destructor*. While there are many similarities, such as the use of the same stressors, between this study and that of Morfin et al. [41], they differ in that the bees were exposed to the stressors for a longer period of time (21 versus 14 days), and the bees in this study were trained for associative learning in the PER assays, which is known to affect gene expression in honey bee brains [40].

*AmAChE-2* expression was also analyzed in this study as it had been shown that acetylcholinesterase activity decreases in bees after associative learning training trials [36]. The pattern of expression of *AmAChE-2* in bees exposed to clothianidin alone was similar to that of *AmNrx-1* and *AmNlg-1*, except that *AmAChE-2* was down-regulated by the highest dose of clothianidin. However, the expression pattern with *V. destructor* alone or combined with clothianidin was quite different for *AmAChE-2.* Unexpectedly, the expression of *AChE-2* decreased in bees only when parasitized by *V. destructor* and exposed to 9 × 10^-4^ ng of clothianidin. In contrast, Morfin et al. [41] found that *AmAChE-2* expression was down-regulated in bees parasitized by *V. destructor* alone. The difference compared to this study could be due to the same reasons as those previously explained for *AmNrx-1* and *AmNlg-1* expression.

The patterns of expression of the three neural-related genes used in this study could not explain by themselves the reduction in memory detected by the PER assay related to the clothianidin dose. However, the use of high throughput techniques, such as RNA sequencing, would be able to examine a wide range of biological pathways related with neural and other processes that could affect learning and memory in honey bees. Further, studies on the effect of neurotoxic insecticides and parasites on the processing centers of the honey bee brain and sensory perception would help better understand the effects of the stressors on odor perception, which could impact odor learning by honey bees.

## 5. Conclusions

None of the stressors affected memory in the short term, but clothianidin, alone or combined with *V. destructor,* affected it in the midterm. However, long-term memory was most sensitive being affected by the two stressors, both alone or combined. Further, an interaction between the stressors was observed in the expression of neural-related genes, but there was not a clear relationship between changes in their expression and the dose of clothianidin. While a down-regulation of *AmNrx-1* and *AmNlg-1* expression by *V. destructor* could possibly explain this effect on memory retention, further investigations using a broader range of genes are warranted to better understand the molecular basis of their impact on honey bee memory and behaviors.

## Figures and Tables

**Table 1:** Mean consumption of sugar syrup (±S.E.) of bees exposed to sublethal doses of clothianidin and/or *V. destructor*. The bees were treated with clothianidin (0 ng clothianidin/µL, 9 × 10^−4^ ng clothianidin/µL, 4.2 × 10^−3^ ng clothianidin/µL, and 1 × 10^−2^ ng clothianidin/µL) and/or *V. destructor* (Vd) for 14 days. No significant differences were observed based on Kruskal–Wallis test (α of 0.05).

Treatment	Mean Consumption of Sugar Syrup (± S.E.)
0 ng/µL	29.41 ± 0.84 µL
9 × 10^−4^ ng/µL	28.65 ± 0.85 µL
4.2 × 10^−3^ ng/µL	28.53 ± 0.55 µL
1 × 10^−2^ ng/µL	28.85 ± 0.90 µL
0 ng/µL + *V. destructor*	27.64 ± 0.44 µL
9 × 10^−4^ ng/µL + *V. destructor*	27.80 ± 0.55 µL
4.2 × 10^−3^ ng/µL + *V. destructor*	27.89 ± 0.36 µL
1 × 10^−2^ ng/µL + *V. destructor*	29.97 ± 0.63 µL

## Supplementary Materials

The following are available online at https://www.mdpi.com/2079-7737/9/9/237/s1, Table S1: Number of bees positive and negative to the PER assay at 2, 24 and 48 h post-training.
