# Supplementary material for: The Combined Effects of Varroa destructor Parasitism and Exposure to Neonicotinoids Affects Honey Bee (Apis mellifera L.) Memory and Gene Expression
Source: Biology (Basel). 2020 Aug 20;9(9):237. doi: 10.3390/biology9090237 (PMC7565275; doi:10.3390/biology9090237)
Supplement: Supplementary file 1 [file biology-09-00237-s001.pdf]

Table S1. Number of bees positive and negative to the PER assay at 2, 24 and 48 h post training.

	**2 h**		**24 h**		**48 h**	
**Treatment**	**Positive**	**Negative**	**Positive**	**Negative**	**Positive**	**Negative**
0 ng / µl	187	84	161	110	90	181
$9X10^{-4}$ ng / µl	67	125	60	132	45	147
$4.2X10^{-3}$ ng / µl	51	123	40	134	28	146
$1X10_{-2}$ ng / µl	24	63	10	77	10	77
0 ng / µl + *V. destructor*	22	18	12	28	7	33
$9X10^{-4}$ ng / µl + *V. destructor*	9	31	7	33	5	35
$4.2X10^{-3}$ ng /µl + *V. destructor*	4	15	1	18	2	17
$1X10_{-2}$ ng / µl + *V. destructor*	9	32	5	36	3	38
